# Levels of polyunsaturated fatty acids correlate with growth rate in plant cell cultures

**DOI:** 10.1038/srep15207

**Published:** 2015-10-15

**Authors:** Coline Meï, Morgane Michaud, Mathilde Cussac, Catherine Albrieux, Valérie Gros, Eric Maréchal, Maryse A. Block, Juliette Jouhet, Fabrice Rébeillé

**Affiliations:** 1 Laboratoire de Physiologie Cellulaire et Végétale, Unité mixte de recherche 5168 CNRS – CEA – INRA – Université Grenoble Alpes, Institut de Recherche en Sciences et Technologies pour le Vivant, CEA Grenoble, 17 rue des Martyrs, 38054, Grenoble Cedex 9, France

## Abstract

In higher plants, fatty acids (FAs) with 18 carbons (18C) represent about 70% of total FAs, the most abundant species being 18:2 and 18:3. These two polyunsaturated FAs (PUFAs) represent about 55% of total FAs in Arabidopsis cell suspension cultures, whereas 18:1 represents about 10%. The level of PUFAs may vary, depending on ill-defined factors. Here, we compared various sets of plant cell cultures and noticed a correlation between the growth rate of a cell population and the level of unsaturation of 18C FAs. These observations suggest that the final level of PUFAs might depend in part on the rate of cell division, and that FAD2 and FAD3 desaturases, which are respectively responsible for the formation of 18:2 and 18:3 on phospholipids, have limiting activities in fast-growing cultures. In plant cell culture, phosphate (Pi) deprivation is known to impair cell division and to trigger lipid remodeling. We observed that Pi starvation had no effect on the expression of *FAD* genes, and that the level of PUFAs in this situation was also correlated with the growth rate. Thus, the level of PUFAs appears as a hallmark in determining cell maturity and aging.

Glycerolipids are major components of the membrane architecture. These acyl-lipids are diester of fatty acids (FAs) and glycerol, and the FA moieties can be either saturated or unsaturated. In higher plants, the main species of FAs are 16C and 18C, representing respectively about 30 and 70% of total FAs^1^. These FAs are present with various saturation levels, generally displaying none (16:0, 18:0) to three (16:3, 18:3) double bonds for the main species. The most abundant FA species are 18:2 and 18:3, representing about 80% of 18C and nearly 55% of total FAs in Arabidopsis cell suspension cultures, whereas 18:1 represents about 10% of total FAs^1^.

Desaturation of 18:0 to 18:1 is catalysed by a soluble desaturase in the stroma using 18:0-ACP as substrate, and a still poorly characterised membrane bound desaturase in the cytosol using 18:0-CoA as substrate^2^. Desaturations of 18:1 to 18:2 then 18:3 are catalysed by four integral membrane FA desaturases (FAD)^2^,^3^. In plastids, these two sequential reactions are catalysed by FAD6 (an ω-6 desaturase) and FAD7 (an ω-3 desaturase) and the desaturation reaction occurs on the acyl linked to the membrane lipid, mono- or digalactosyl diacylglycerol (MGDG or DGDG), phosphatidylglycerol and sulfoquinovosyldiacylglycerol^4^. In the endoplasmic reticulum (ER), equivalent reactions are respectively catalysed by FAD2 and FAD3 on phospholipids^3^,^5^,^6^. A recent study has shown that FAD6 and FAD7 on the one hand, and FAD2 and FAD3 on the other hand, can physically associate via protein-protein interactions, providing a metabolic channeling from 18:1 to 18:3^7^. In such a situation 18:2 would not be released as an intermediary product, and therefore should be kept low in the whole FA composition. Since it is apparently not the case, at least in Arabidopsis^8^,^9^, it is possible that the two desaturases also operate independently, or that FAD2 or FAD6 are in excess compared to FAD3 or FAD7 respectively. In the endoplasmic reticulum, phosphatidylcholine (PC) plays a major role in these reactions because it is the site of ‘acyl editing’, a process involving desaturation of the acyl groups followed by a rapid deacylation-reacylation cycle that exchanges FAs from PC with FAs from the acyl-CoA pool^10^. Through this cycle, 18:1-CoA is attached to PC by a lyso-phosphatidylcholine acyltransferase (LPCAT), before its conversion to 18:2 and 18:3 by FAD2 and FAD3. These PUFAs are thereafter released from PC in the acyl-CoA pool by phospholipase A cleavage/acyl-CoA synthetase or the reverse action of LPCAT, to be eventually available for additional glycerolipid syntheses. The rate of this cycle has been estimated to be significantly higher than the rate of FA synthesis in developing soybean embryos, which should limit the accumulation of relatively saturated membrane lipid species^11^.

Unsaturated fatty acids play key roles in membrane structure and function. Indeed, the degree of saturation not only impacts the physicochemical characteristics of the FA, such as the melting point or the viscosity^12^, but also the molecular shape of the lipid to which it is attached^13^. This, in turn, influences the membrane lipid behavior and the bilayer packing. For instance, saturated phosphatidylethanolamines (PE) form a lamellar phase whereas unsaturated PE form an hexagonal HII (inverted non lamellar) phase^14^,^15^. Consequently, the enzymes involved in the formation of unsaturated fatty acids, play major roles in membrane formation, function and integrity.

In addition, the unsaturated level of FAs in plants may vary, at least in a certain range, in response to different environmental situations. Related to the fact that FAD desaturations are oxygen-dependent reactions, it has been observed that the aeration conditions, i.e. the concentration of dissolved oxygen in the cytosol, affect the FA unsaturation pattern of lipids^16^,^17^. From a physiological point of view, desaturases are also important to maintain cellular function and plant viability in specific conditions, such as cold^9^ or salt^18^ stresses. Cold-induced change of the FA unsaturation level is well documented and is generally explained by the fact that freezing tolerance requires a higher unsaturation level to keep a correct membrane fluidity^9^,^19^. As an example, a *fad2* mutant could not grow, and subsequently died when cultured at a low temperature (6 °C), indicating that PUFAs are essential to maintain cellular viability in cold conditions^20^. In addition to transcriptional controls^5^,^18^, post-translational regulations of these desaturase activities might also contribute to adjust the desaturation level in plants. From this point of view, it was shown that FAD2^21^ and FAD3^22^ are less subjected to degradation at low temperatures, a process controlled by the proteasomal pathway that might play a role in this adaptative response.

Beside these known environmental effects on the unsaturation level of FAs, other ill-defined factors may alter the level of PUFAs. This is apparent with cell suspension cultures where the extent of PUFAs may vary from one culture to another, making the comparison of results sometimes difficult. In an attempt to better understand what triggers such variations, we measured the impact of growth rate upon the distribution of PUFAs in our plant models. Using cell suspension cultures that mainly contain phospholipids, we observed a correlation between the growth rate and the level of unsaturation of 18C FAs, suggesting that FAD2 and FAD3 desaturases might have limiting activities in fast growing cultures and that the final level of unsaturation might also depend on the rate of division of the plant cell population.

## Results

To study lipid metabolism in higher plants, three models of cell suspension cultures were used: stem cells of *Acer pseudoplatanus* (Sycamore), leaf meristem cells of *Arabidopsis thaliana* (Arabidopsis) and callus cultures of meristem cells of Arabidopsis. In these cultures, membrane lipids are mainly phospholipids (80 to 90%) with only low amount of galactolipids (10 to 20%)^23^. Indeed, these cells are growing either heterotrophically (Sycamore cells, Arabidopsis calli) or mixotrophically (Arabidopsis cells) and display only amyloplasts (Sycamore cells and Arabidopsis calli) or poorly developed thylakoid membranes with low amount of chlorophyll (Arabidopsis cells). This suggests that PUFAs in our plant models are mostly generated by the FAD2/FAD3 system in the ER, with a minor but significant contribution of the FAD6/FAD7 couple in plastids. This composition is highly different in leaves where galactolipids represent more than 60% of glycerolipids^24^, suggesting that FAD2 and FAD3 might not be the main providers of PUFAs in these tissues.

**Figure 1:**
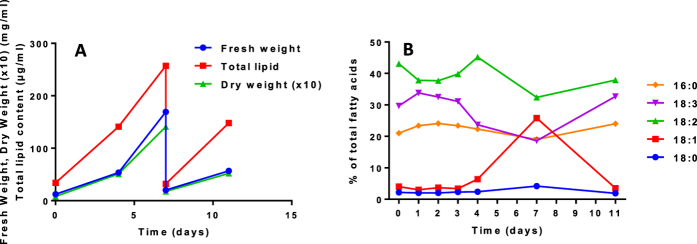
Fresh weight and dry weight evolutions and lipid distribution in Sycamore cells during the course of a culture cycle. (**A**) representative experiment showing the fresh weight and the lipid content evolutions after the transfer of the cells in a fresh media. At day 7, an aliquot of the cell suspension was transferred in a new fresh media for a new cycle of growth. Each day, an aliquot was withdrawn for fatty acid analyses. (**B**) main FA distribution in Sycamore cells cultivated as shown in (A).

When using higher plant cell suspension cultures, there are variations in the growth rate, even in standard controlled conditions, which makes it sometimes difficult to compare cultures that grew at different periods. There is no clear explanation to these fluctuations, although it is likely that slight changes in the density of the initial inoculum, which can vary in the range of plus or minus 10%, may impact the growth rate by modulating the length of the initial lag phase of the culture cycle. However, these variations might also be a source of information, and we looked for a possible correlation between the growth rate and the variability of the FA unsaturation level we often observed in these cultures. In the representative experiment shown in Fig. 1, Sycamore cells displayed alternatively slow and rapid periods of growth as estimated through the variations of either the fresh weight or the dry weight. At the beginning of the experiment, just after the transfer in a new medium, cells were dividing rather slowly during the initial lag phase, then more rapidly during the exponential phase of growth, then slowly again during the second lag phase following the second transfer and dilution into a fresh medium (Fig. 1A). During these growth phases, the amount of lipids increased roughly in parallel with the biomass. The profile of FAs was determined along the culture period (Fig. 1B). 18C, but not 16C, FA species displayed significant changes during the exponential phase of growth: 18:3 and to a lower extent 18:2 decreased whereas 18:1 strongly increased, suggesting that the rate of desaturation of 18:1 was reduced during the fast-growing period.

**Figure 2:**
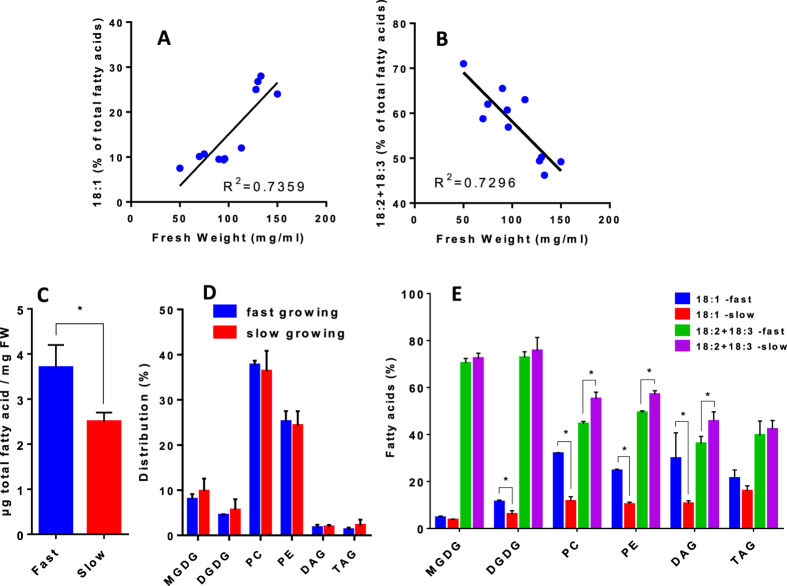
Correlation curves between the growth rate of Arabidopsis cells and the unsaturation levels of 18C FA species. (**A**) correlation curve between the proportion of 18:1 and the fresh weight of cell suspensions measured after 3 days of culture. (**B**) correlation curve between the proportion of PUFAs (18:2+18:3) and the fresh weight of cell suspensions measured after 3 days of culture. The initial fresh weight at the beginning of the experiment was 30 ± 5 mg/ml for each culture. Correlation factors R^2^ were calculated by linear regression using GraphPad Prism software. (**C**) total amount of FAs in either fast or slow growing cells. (**D**) distribution of the main polar glycerolipids (MGDG and DGDG for main galactolipids; PC and PE for main phospholipids), and neutral lipids (DAG and TAG) in fast and slow growing cells. (**E**) proportion of 18:1 and PUFAs in major glycerolipids (MGDG, DGDG, PC and PE) and in neutral lipids (DAG and TAG) from either fast or slow growing cells. The data are means ± SD of three biological repeats for each condition. Statistical significant differences (P < 0.05) are shown by an asterisk and were calculated by a multiple t test using GraphPad Prism software.

We therefore sought to correlate in Arabidopsis the growth rate in standard complete medium with the 18C unsaturation level, exploiting the variability that occurs between different culture cycles. In Fig. 2A,B, we plotted the unsaturated level of 18C versus the growth rate for more than 10 different cultures grown in the same environment and harvested 3 days after renewing the media. We observed a significant correlation between the growth rate and the proportions of 18:1 and 18:2+18:3: with higher growth rates there were higher levels of 18:1 and lower levels of 18:2+18:3. Detailed analyses of the lipid composition in fast growing and slow growing cell cultures show that slow growing cells displayed 25–30% less lipids, but an unchanged distribution of the main glycerolipids (Fig. 2C,D). However, the levels of 18:1 and PUFAs were modified in PC and PE as it was observed in the total FA analysis (Fig. 2E). Diacylglycerols (DAGs) also were affected but not triacylglycerols (TAGs), suggesting that the synthesis of TAGs from DAG backbones favor PUFA rich species. The fact that DAG and PC were similarly affected illustrates the dynamic acyl exchange between these two lipids via the phosphatidylcholine:diacylglycerol choline phosphotransferase (PDCT). Together with acyl editing, this process might also play a central role in the regulation of glycerolipid desaturation^25^. Interestingly, the PUFA levels in galactolipids (MGDG and DGDG) did not change between the two situations, although the DGDG level of 18:1 was slightly increased in the fast growing condition. This slight increase could result from a eukaryotic origin of the glycerol backbone, since a fraction of the DGDG molecules are formed from ER-derived DAGs^26^. Altogether, these results suggest that the FAD6/FAD7 system was less impacted in fast growing cells than FAD2/FAD3.

**Figure 3:**
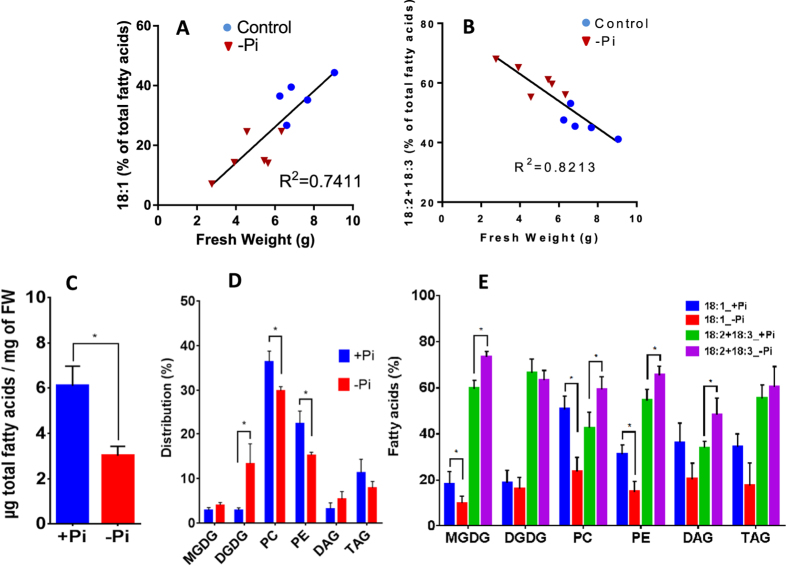
Correlation curves between the growth rate of Arabidopsis calli grown in a control medium or in a medium devoid of Pi and the unsaturation levels of 18C FA species. Fresh weight and fatty acid distribution were measured after 8 days of growth. The initial fresh weight was 1.5 ± 0.2 g for each culture. (**A**) correlation curve between the proportion of 18:1 and the fresh weight of Arabidopsis calli. (**B**) correlation curve between the proportion of PUFAs (18:2+18:3) and the fresh weight of Arabidopsis calli. Correlation factors R^2^ were calculated by linear regression using GraphPad Prism software. (**C**) total amount of FAs in cultures grown either in presence or in absence of Pi. (**D**) distribution of the main polar glycerolipids (MGDG and DGDG for main galactolipids; PC and PE for main phospholipids), and neutral lipids (DAG and TAG) in cultures grown either in presence or in absence of Pi. (**E**) proportion of 18:1 and PUFAs in major glycerolipids (MGDG, DGDG, PC and PE) and in neutral lipids (DAG and TAG) from cultures grown either in presence or in absence of Pi. The data are means ± SD of three biological repeats for each condition. Statistical significant differences (P < 0.05) are shown by an asterisk and were calculated by a multiple t test using GraphPad Prism software.

**Figure 4:**
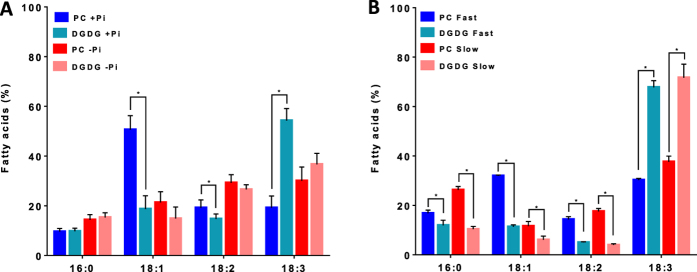
FA distribution in PC and DGDG. Detail distribution of FAs of PC and DGDG in Arabidopsis calli grown with (+Pi) or without (−Pi) phosphate (**A**) or in Arabidopsis cell suspension cultures displaying fast or slow growing rates (**B**). The data are means ± SD of three biological repeats for each condition. Statistical significant differences (P < 0.05) are shown by an asterisk and were calculated by a multiple t test using GraphPad Prism software.

To confirm the relationship between desaturation of 18:1 and growth rate, we compared the FA profile of Arabidopsis calli grown in liquid suspension either in the presence or in the absence of Pi (Fig. 3). As previously observed^23^, phosphate starvation induced a significant reduction of the growth rate (Fig. 3A,B) associated with lipid remodeling, as shown by a fourfold increase of DGDG and a 20% decrease of phospholipids (Fig. 3D). Indeed, plants adapt to such a situation by replacing part of their phospholipids by galactolipids, mainly DGDG. Despite these changes, phospholipids in Pi-deprived calli still represent more than 60% of all glycerolipids. In our conditions (no more than 8 days of Pi starvation), there was a decrease of the total FAs content and no accumulation of TAG (Fig. 3C,D). TAG accumulation is a late response to strong Pi deprivation in higher plants^25^ and microalgae^27^, seen after 13 days or more of Pi starvation. As for Arabidopsis cell suspension cultures, there was a significant correlation between the growth rate (limited or not by the absence of Pi) and either the level of 18:1 or the level of PUFAs (18:2+18:3), both levels varying in opposite directions (Fig. 3A,B). Detailed analyses of the lipid composition between control and Pi-starved cultures show that the main phospholipids were affected in their 18:1 and PUFA compositions (Fig. 3E). Here again, low rates of growth (Pi-starvation) induced an increase of PUFAs in phospholipids and DAGs, whereas these effects were attenuated in TAGs (Fig. 3E). The striking difference arises from galactolipids which play a central role in this low-Pi-induced lipid remodeling. Indeed, the synthesis of DGDG during Pi starvation is mainly achieved at the surface of plastids by MGD2/MGD3 and DGD2 enzymes^26^,^28^, through a transitory pool of MGDG. MGD2 and MGD3 preferentially use DAG backbones from the ER-derived pathway, probably arising from phospholipid degradation^29^. Thus, the pool of MGDG in the Pi-limiting situation is a mixture of molecules with glycerol backbones originating from both the prokaryotic and the eukaryotic pathways, which might explain the observed changes in the unsaturation level (decrease of 18:1 and increase of PUFAs, Fig. 3E). Although the unsaturation level of DGDG did not appear to be modified, it must be kept in mind that the FA composition in DGDG is quite different in the two situations^23^. In Pi-starved cultures, but not in control cultures, DGDG is mainly built from DAG backbones originating from PC^29^, and so its FA composition resembled much the one of PC (Fig. 4A). This is not the case when fast and slow growing cell suspension cultures are compared (Fig. 4B), indicating that changes in the FA composition in MGDG and DGDG were a direct consequence of Pi deprivation. This is a complex situation, not yet fully understood, and it is still not known how extraplastidial DGDG is metabolized and if it is edited as PC is.

**Figure 5:**
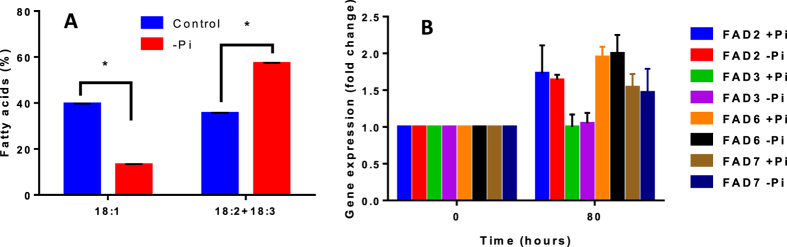
Effect of a Pi deficiency on the expression levels of *FAD2*, *FAD3, FAD6* and *FAD7* genes in Arabidopsis cell suspension cultures. (**A**) 18:1 and PUFA (18:2+18:3) distributions in cells cultivated for 80 h in control medium or in a medium devoid of Pi. (**B**) qPCR determination of the expression levels of *FADs* in cells grown for 80 h either in a control medium or in a medium devoid of Pi. Results are expressed as fold changes versus the initial control conditions. The data are means ± SD of three biological repeats for each condition. Statistical significant differences (P < 0.05) are shown by an asterisk and were calculated by a multiple t test using GraphPad Prism software.

Similar effects of Pi starvation were also seen in cell suspension cultures. Indeed, as shown in Fig. 5A, Arabidopsis cells grown in a medium devoid of Pi for 80 h exhibited higher 18:2+18:3 and lower 18:1 contents, supporting the above results. To check whether the increase of PUFAs in Pi-deficient cells could be attributed to an increase of *FAD* expression, we measured the effect of Pi starvation on *FAD*2, *FAD*3, *FAD*6 and *FAD*7 transcript levels. No difference in *FAD* expression levels could be observed between the two growth conditions (Fig. 5B), indicating that Pi starvation did not impact the expression of these four genes, as previously observed^30^. These results suggest that the rate at which FAs were desaturated was slower than the rate at which cells divided and lipids were synthesised, at least in fast growing conditions. In other words, desaturase activities (essentially FAD2 and FAD3) leading to 18:3 from 18:1 were probably limiting, and the final level of unsaturation could partly depend on the rate of FA synthesis and membrane turnover.

## Discussion

The analyses presented here strongly suggest that a negative correlation occurs between the growth rate of a plant cell culture and the level of PUFA (18:2+18:3). In fast-growing tissues (calli) or in highly dividing cells (cell suspension cultures), the proportion of PUFA is lower than in slow growing ones. It is noteworthy that phospholipids were the main lipids affected by these changes, which points out a particular sensitivity of the FAD2/FAD3 system to the growth condition. It is difficult to draw conclusions about the FAD6/FAD7 system because galactolipid remodeling in the Pi-starved situation is rather complex and involves an increased recruitment of glycerol backbones from an ER origin. However, the experiments with the fast and slow growing cell suspension cultures suggest that FAD6/FAD7 were less affected by the growth rate. Whether this difference results from the low amount of plastidial lipids present in our plant model remains an open question.

It might be possible that in situations where membrane syntheses and/or turnover are fast, the activities of FAD2/FAD3 are not high enough to match phospholipid synthesis and editing, leading to 18:1 accumulation in membrane lipids. In developing soybean embryos it was shown that the rate of PC editing was higher than the rate of FA synthesis^11^. However the rate of lipid synthesis in soybean is slow compared to other oilseed plants^11^ and the situation may differ in other plants. When considering a whole plant, the situation may also vary from one tissue to another, depending on the dividing activity of the tissue. In the cell cultures analysed here, the amount of 18:1 dropped when the rate of division decreased (see Fig. 1) indicating that FAD activities were not or less limiting in this condition.

These results also indicate that the level of unsaturation can fluctuate from one culture to another, these variations resulting from even small differences in the growth rate. Thus, every treatment that impacts the growth rate or cell division could affect the unsaturation level, unless it also affects the rate of lipid synthesis and/or the expression or regulation of FADs. In Pi-limited higher plants, growth and FA production are strongly reduced^23^. Pi limitation did not affect the gene expression of *FAD2*, *FAD3*, *FAD6* and *FAD7*, and the calculated correlation factors between the global unsaturation level and the growth rate were similar to those obtained in complete medium growth conditions (compared Fig. 2 and 3). Thus, this study suggests that the desaturation of lipids by FAD2 and FAD3 in fast dividing systems might not reach a steady state and might be subjected to variations dictated by the growth rate. The *in vivo* correlation between FAD2/FAD3 activities and cell proliferation rates is therefore also defining the level of PUFAs as a hallmark of cell maturity and aging in plants.

**Figure 6:**
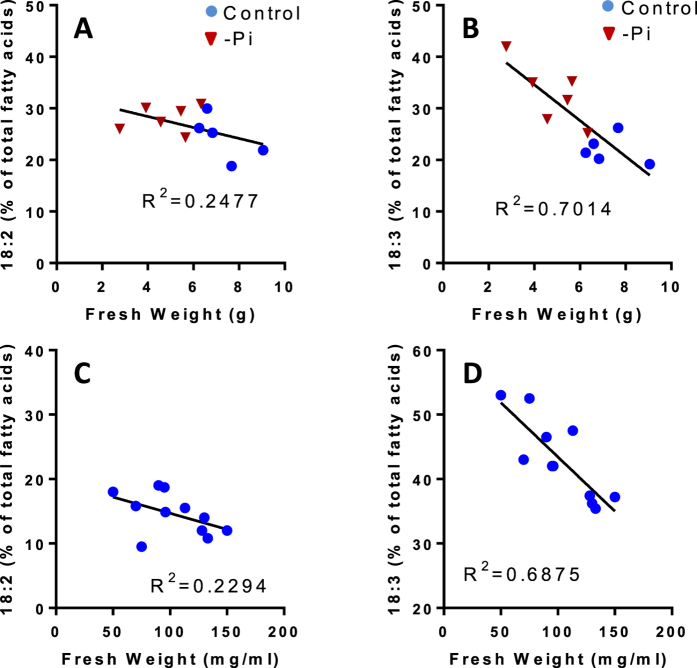
Relationships between the growth rate of Arabidopsis calli or Arabidopsis cells and the distribution levels of 18:2 and 18:3. (**A**) correlation curve between 18:2 and fresh weights of calli grown in liquid medium with or without Pi. (**B**) same as A, but for 18:3. The initial fresh weight was 1.5 ± 0.2 g for each culture and the biomass evolution was measured after 8 days of growth. (**C**) correlation curve between 18:2 and fresh weights of cell suspension cultures. (**D**) same as C, but for 18:3. The biomass was measured after 3 days of culture, and the initial fresh weight at the beginning of the experiment was 30 ± 5 mg/ml. Correlation factors R^2^ were calculated by linear regression using GraphPad Prism software.

The question of how these desaturases are operating and are regulated *in vivo* remains open. Interestingly, in these experiments we often observed less variation with 18:2 than with 18:1 and 18:3 (see Fig. 1B). Indeed, with Arabidopsis calli and cell suspension cultures there was no significant correlation between the proportion of 18:2 and the growth rate, in contrast to what is observed with 18:3 (Fig. 6A,B). The fact that the proportion of 18:2 fluctuates less than the proportions of 18:1 and 18:3 supports either the hypothesis of a channeling from 18:1 to 18:3 where 18:2 is not systematically released in the bulk medium, or the hypothesis of a limiting activity of FAD2 compared to FAD3. In the first hypothesis, if channeling is the major process for 18:1 desaturation, the 18:2 level should remain quite low, unless FAD2 is in excess compared to FAD3. In our models, 18:2 is not maintained at a low level since in non-photosynthetic Sycamore cells and in Arabidopsis cells it represents respectively 40% and 10–15% of total FAs. Otherwise, if the two desaturases operate independently, it is also possible that FAD2 activity is more limiting than FAD3 activity, as it was suggested for Flax^31^. It is clear that a better knowledge of the stoichiometry and the kinetic parameters of these two enzymes would help to better understand how these PUFAs equilibrate and fluctuate in plant cells.

## Methods

### Cell cultures

*Acer pseudoplatanus* cells were grown as suspension cultures in a medium previously defined^32^. Cells were maintained as 250 ml cultures on a vertical rotational shaker (125 rpm) at 22 °C. Cells were subcultured every 7 days, as previously described^23^. At each time point, about 0.4 g of cells were collected, filtered to remove the medium, weighted and frozen in liquid nitrogen for lipid analyses.

*Arabidopsis thaliana* (ecotype Columbia) cells were grown as suspension cultures (200 mL) in Murashige and Skoog medium (MSP09-50LT, Caisson Laboratories, Inc, USA) devoid or not of phosphate (Pi), and supplemented with 1.5% (w/v) sucrose, 1.2 mg l^−1^ 2,4-dichlorophenoxyacetic acid, potassium phosphate monobasic 50 mg l^−1^, and potassium phosphate dibasic 39 mg l^−1^. Cultures were kept under continuous light (100 μE m^−2^ s^−1^) at 22 °C, and agitated with rotary shaking at 125 rpm. Cells were subcultured every 7 days with an initial cell density of 30 mg mL^−1^. At each time point, about 0.5 g of cells were collected, filtered to remove the medium, weighted and frozen in liquid nitrogen for lipid analyses.

*Arabidopsis thaliana* (ecotype Columbia) calli were obtained from mesophyll tissue of two week old Arabidopsis rosette leaves, and grown on agar plates containing Murashige and Skoog medium (MSP09-50LT, Caisson Laboratories, Inc, USA) supplemented with 3% (w/v) sucrose, 1.2 mg L^−1^ 2,4-dichlorophenoxyacetic acid, potassium phosphate monobasic 50 mg l^−1^, and potassium phosphate dibasic 39 mg l^−1^. For each experiment, about 1.5 g of calli were withdrawn from the agar plate and suspended in liquid Murashige and Skoog medium devoid or not of Pi (Caisson Laboratories, Inc, USA), supplemented with 1.5% (w/v) sucrose and 1.2 mg l^−1^ 2,4-dichlorophenoxyacetic acid. At each time point, aliquots were collected from the callus suspension cultures, filtered to remove the medium, and the calli were weighted and frozen in liquid nitrogen for lipid analyses.

### Lipid analyses

Freeze-dried cells were suspended in 4 mL of boiling ethanol for 5 minutes to prevent lipid degradation, and lipids were extracted by addition of 2 mL methanol and 8 mL chloroform at room temperature^27^. The mixture was then saturated with argon and kept for 1 hour at room temperature. After filtration through glass wool, cell debris were rinsed with 3 mL chloroform/methanol 2:1, v/v, and 5 mL of NaCl 1% were then added to the filtrate to initiate biphase formation. The chloroform phase was collected and dried under argon before solubilizing the lipid extract in pure chloroform. Total glycerolipids were quantified from their FAs after transformation as methyl esters (FAME), and analysed by a gas chromatography-flame ionization detector (GC-FID) (Perkin Elmer) on a BPX70 (SGE) column, as thoroughly described elsewhere^27^. FAME were identified by comparison of their retention times with those of standards (Sigma) and quantified by the surface peak method using 15:0 for calibration. Extraction and quantification were performed from at least 3 biological repeats. To quantify the various classes of glycerolipids, lipids were separated by thin layer chromatography (TLC) onto glass-backed silica gel plates (Merck) using two distinct resolving systems for polar and neutral lipids, as previously described^27^. Lipids were then visualized under UV light, after spraying with 2% 8-anilino-1-naphthalenesulfonic acid in methanol, and scraped off the plate. Lipids were recovered from the silica powder after addition of 1.35 mL chloroform:methanol 1:2 v/v, thorough mixing and addition of 0.45 mL chloroform and 0.8 mL H2O and collection of the chloroform phase^33^. Lipids were then dried under argon and quantified by methanolysis and GC-FID as described above.

### qPCR

Real-time quantitative RT-PCR experiments were performed using cDNA synthesised from total RNA isolated from control and treated Arabidopsis cells (Reverse-it first strand synthesis kit, ABgene). Amplification of contaminating DNA was prevented by DNAse treatment of RNA samples. Specific primer sequences designed for *FAD2* (At3g12120), *FAD3* (At2g29980), *FAD6* (At4g30950) and *FAD7* (At3g11170) genes were: GCC TTG GTA TAG AGG CAA GGA ATG G (*FAD3*, Fw); TGC TTT CGT GGG GTC GAC CA (*FAD3*, Rv);.CAA TGA CCG AGA ACG CCT CC (*FAD2*, Fw); GGC AAC GAG GGA TGA GTG TG (*FAD2*, Rv); TCT GCT ACC GTT GGC TTG GGC (*FAD6*, Fw); TTT TGG CGT GAT GGC GGT CGT (*FAD6*, Rv); CGA CCT CTC CCC AGA ATC TAC ACA (*FAD7*, Fw); GGT GTG CTC ACA TTC AAC GCC CA (*FAD7*, Rv). The real-time PCR reactions were carried out on a Rotor-Gene 3000 instrument (Corbett Research) using SYBR Green JumpStart Taq ReadyMix (Sigma-Aldrich). Quantification of gene expression was performed using the comparative C_T_ method with the Rotor-Gene 3000 Software. Each data were normalised with 3 reference genes chosen for their absence of variation in Pi limiting conditions (*ACT8* -At1g49240; *UBQ10* -At4g05320; *TIP41-like* -At4g34270). The amplification efficiencies of these oligo pairs was close to 1 (0.9–1.1). Each value represents the average of three biological repeats, each repeat being analysed in triplicate. Statistical analyses were done using GraphPad Prism software.

## Additional Information

**How to cite this article**: Meï, C. *et al.* Levels of polyunsaturated fatty acids correlate with growth rate in plant cell cultures. *Sci. Rep.* **5**, 15207; doi: 10.1038/srep15207 (2015).

## References

[b1] Tjellstroem H., Yang Z., Allen D. K. & Ohlrogge J. B. Rapid kinetic labeling of Arabidopsis cell suspension cultures: implications for models of lipid export from plastids. Plant Physiology 158, 601–611 (2012). 22128138 10.1104/pp.111.186122 PMC3271753

[b2] Shanklin J. & Cahoon E. B. Desaturation and related modifications of fatty acids. Annual Review of Plant Physiology and Plant Molecular Biology 49, 611–641 (1998). 10.1146/annurev.arplant.49.1.611 15012248

[b3] Dolch L. J. & Marechal E. Inventory of fatty fcid fesaturases in the pennate fiatom Phaeodactylum tricornutum. Mar Drugs 13, 1317–1339 (2015). 25786062 10.3390/md13031317 PMC4377986

[b4] Joyard J. *et al.* Chloroplast proteomics highlights the subcellular compartmentation of lipid metabolism. Prog Lipid Res 49, 128–158 (2010). 19879895 10.1016/j.plipres.2009.10.003

[b5] Okuley J. *et al.* Arabidopsis FAD2 gene encodes the enzyme that is essential for polyunsaturated lipid synthesis. Plant Cell 6, 147–158 (1994). 7907506 10.1105/tpc.6.1.147 PMC160423

[b6] Arondel V. *et al.* Map-Based cloning of gene controlling omega-3-fatty-acid desaturation in Arabidopsis. Science 258, 1353–1355 (1992). 1455229 10.1126/science.1455229

[b7] Lou Y., Schwender J. & Shanklin J. FAD2 and FAD3 desaturases form heterodimers that facilitate metabolic channeling *in vivo*. Journal of Biological Chemistry 289, 17996–18007 (2014). 24811169 10.1074/jbc.M114.572883 PMC4140268

[b8] Song W., Maeda H. & DellaPenna D. Mutations of the ER to plastid lipid transporters TGD1, 2, 3 and 4 and the ER oleate desaturase FAD2 suppress the low temperature-induced phenotype of Arabidopsis tocopherol-deficient mutant vte2. Plant Journal 62, 1004–1018 (2010). 20345604 10.1111/j.1365-313X.2010.04212.x

[b9] Chen M. & Thelen J. J. ACYL-LIPID DESATURASE2 is required for chilling and freezing tolerance in Arabidopsis. Plant Cell 25, 1430–1444 (2013). 23585650 10.1105/tpc.113.111179 PMC3663278

[b10] Bates P. D. *et al.* Acyl editing and headgroup exchange are the major mechanisms that direct polyunsaturated fatty acid flux into triacylglycerols. Plant Physiology 160, 1530–1539 (2012). 22932756 10.1104/pp.112.204438 PMC3490606

[b11] Bates P. D., Durrett T. P., Ohlrogge J. B. & Pollard M. Analysis of acyl fluxes through multiple pathways of triacylglycerol synthesis in developing soybean embryos. Plant Physiology 150, 55–72 (2009). 19329563 10.1104/pp.109.137737 PMC2675710

[b12] Knothe G. “Designer” biodiesel: optimizing fatty ester composition to improve fuel properties. Energy & Fuels 22, 1358–1364 (2008).

[b13] Lindblom G., Oradd G., Rilfors L. & Morein S. Regulation of lipid composition in Acholeplasma laidlawii and Escherichia coli membranes: NMR studies of lipid lateral diffusion at different growth temperatures. Biochemistry 41, 11512–11515 (2002). 12234195 10.1021/bi0263098

[b14] Seddon J. M. Structure of the inverted hexagonal (HII) phase, and non-lamellar phase-transition of lipids. Biochimica Et Biophysica Acta 1031, 1–69 (1990). 2407291 10.1016/0304-4157(90)90002-t

[b15] Jouhet J. Importance of the hexagonal lipid phase in biological membrane organization. Frontiers in Plant Science 4 (2013). 10.3389/fpls.2013.00494 PMC3848315 24348497

[b16] Rebeille F., Bligny R. & Douce R. Oxygen and Temperature effects on the fatty-acid composition in sycamore cells (Acer-Pseudoplatanus L.). Biochimica Et Biophysica Acta 620, 1–9 (1980). 7417473 10.1016/0005-2760(80)90178-2

[b17] Bligny R., Rebeille F. & Douce R. O-2-Triggered changes of membrane fatty-acid composition have no effect on arrhenius discontinuities of respiration in Sycamore (Acer-Pseudoplatanus L.) cells. Journal of Biological Chemistry 260, 9166–9170 (1985). 4019468

[b18] Zhang J. *et al.* Arabidopsis fatty acid desaturase FAD2 is required for salt tolerance during seed germination and early seedling growth. PLoS One 7, 1–12 (2012). 10.1371/journal.pone.0030355 PMC3261201 22279586

[b19] Murata N. & Wada H. Acyl-lipid desaturases and their importance in the tolerance and acclimatization to cold of cyanobacteria. Biochemical Journal 308, 1–8 (1995). 7755550 10.1042/bj3080001 PMC1136835

[b20] Miquel M., James D., Dooner H. & Browse J. Arabidopsis requires polyunsaturated lipids for low-temperature survival. Proceedings of the National Academy of Sciences of the United States of America 90, 6208–6212 (1993). 11607410 10.1073/pnas.90.13.6208 PMC46897

[b21] Tang G. Q., Novitzky W. P., Griffin H. C., Huber S. C. & Dewey R. E. Oleate desaturase enzymes of soybean: evidence of regulation through differential stability and phosphorylation. Plant Journal 44, 433–446 (2005). 16236153 10.1111/j.1365-313X.2005.02535.x

[b22] O’Quin J. B. *et al.* Temperature-sensitive post-translational regulation of plant omega-3 fatty-acid desaturases is mediated by the endoplasmic reticulum-associated degradation pathway. Journal of Biological Chemistry 285, 21781–21796 (2010). 20452984 10.1074/jbc.M110.135236 PMC2898375

[b23] Jouhet J., Marechal E., Bligny R., Joyard J. & Block M. A. Transient increase of phosphatidylcholine in plant cells in response to phosphate deprivation. FEBS Lett. 544, 63–68 (2003). 12782291 10.1016/s0014-5793(03)00477-0

[b24] Li-Beisson Y. *et al.* Acyl-lipid metabolism. The Arabidopsis book/American Society of Plant Biologists 11, e0161–e0161 (2013). 23505340 10.1199/tab.0161 PMC3563272

[b25] Pant B. D. *et al.* The transcription factor PHR1 regulates lipid remodeling and triacylglycerol accumulation in Arabidopsis thaliana during phosphorus starvation. Journal of Experimental Botany 66, 1907–1918 (2015). 25680792 10.1093/jxb/eru535 PMC4378627

[b26] Kelly A. A. & Dormann P. Green light for galactolipid trafficking. Curr Opin Plant Biol 7, 262–269 (2004). 15134746 10.1016/j.pbi.2004.03.009

[b27] Abida H. *et al.* Membrane glycerolipid remodeling triggered by nitrogen and phosphorus starvation in Phaeodactylum tricornutum. Plant Physiology 167, 118–136 (2015). 25489020 10.1104/pp.114.252395 PMC4281014

[b28] Hartel H., Dormann P. & Benning C. DGD1-independent biosynthesis of extraplastidic galactolipids after phosphate deprivation in Arabidopsis. Proceedings of the National Academy of Sciences of the United States of America 97, 10649–10654 (2000). 10973486 10.1073/pnas.180320497 PMC27079

[b29] Awai K. *et al.* Two types of MGDG synthase genes, found widely in both 16:3 and 18:3 plants, differentially mediate galactolipid syntheses in photosynthetic and nonphotosynthetic tissues in Arabidopsis thaliana. Proc. Natl. Acad. Sci. USA 98, 10960–10965 (2001). 11553816 10.1073/pnas.181331498 PMC58581

[b30] Morcuende R. *et al.* Genome-wide reprogramming of metabolism and regulatory networks of Arabidopsis in response to phosphorus. Plant Cell Environ. 30, 85–112 (2007). 17177879 10.1111/j.1365-3040.2006.01608.x

[b31] Fofana B., Cloutier S., Duguid S., Ching J. & Rampitsch C. Gene expression of stearoyl-ACP desaturase and Delta 12 fatty acid desaturase 2 is modulated during seed development of flax (Linum usitatissimum). Lipids 41, 705–712 (2006). 17069354 10.1007/s11745-006-5021-x

[b32] Bligny R. Growth of suspension-cultured Acer-Pseudoplatanus L cells in automatic culture units of large volume. Plant Physiology 59, 502–505 (1977). 16659880 10.1104/pp.59.3.502 PMC542431

[b33] Bligh E. G. & Dyer W. J. A rapid method of total lipid extraction and purification. Can. J. Biochem. Physiol. 37, 911–917 (1959). 13671378 10.1139/o59-099

